# Cancer Stem Cells: Metabolic Characterization for Targeted Cancer Therapy

**DOI:** 10.3389/fonc.2021.756888

**Published:** 2021-11-05

**Authors:** Jasmeet Kaur, Shalmoli Bhattacharyya

**Affiliations:** Department of Biophysics, Postgraduate Institute of Medical Education and Research (PGIMER), Chandigarh, India

**Keywords:** metabolism, cancer stem cell, glucose, glutamine, OxPhos

## Abstract

The subpopulation of cancer stem cells (CSCs) within tumor bulk are known for tumor recurrence and metastasis. CSCs show intrinsic resistance to conventional therapies and phenotypic plasticity within the tumor, which make these a difficult target for conventional therapies. CSCs have different metabolic phenotypes based on their needs as compared to the bulk cancer cells. CSCs show metabolic plasticity and constantly alter their metabolic state between glycolysis and oxidative metabolism (OXPHOS) to adapt to scarcity of nutrients and therapeutic stress. The metabolic characteristics of CSCs are distinct compared to non-CSCs and thus provide an opportunity to devise more effective strategies to target CSCs. Mechanism for metabolic switch in CSCs is still unravelled, however existing evidence suggests that tumor microenvironment affects the metabolic phenotype of cancer cells. Understanding CSCs metabolism may help in discovering new and effective clinical targets to prevent cancer relapse and metastasis. This review summarises the current knowledge of CSCs metabolism and highlights the potential targeted treatment strategies.

## Introduction

Cancer causes significant deaths worldwide, despite major innovations in treatment therapy strategies, radiation- and chemo-therapy and drug delivery technologies. A major contributor to the cancer treatment-associated toxicities and resistance (1–3), is their inability to eradicate subset of cancer stem cells (CSCs) which drive tumour growth and heterogeneity. CSCs presence makes tumors resistant to conventional therapies (4). Density of CSCs is a proven prognostic marker in various cancers (5, 6), thus targeting CSCs is an effective way for treating cancer.

CSCs self-renewal and asymmetric division capacity help tumors to regenerate and propagate post-treatment. CSCs populations provide high radio- and chemo-resistance due to efficient DNA repair and cellular redox homeostasis, protective tumor microenvironment, escape from immune response and unique metabolic phenotype (7–10). CSCs use metabolic reprogramming to escape immune system (11) and grant them plasticity (12). Metabolic reprogramming induce M2 phenotype in tumor-associated macrophages (TAMs) (13, 14) and glycolysis induce IL-6 secretion in M2 macrophages (15). Secreted IL-6 promotes CSC phenotype in cancer cells (16) *via* activation of STAT3/NFκB signaling pathways (17). CSCs in turn induce M2 phenotype in TAMs to confer drug resistance and tumorigenicity in CSCs by blocking the anti-tumor CD8^+^ response during chemotherapy (18).

**Figure 1:**
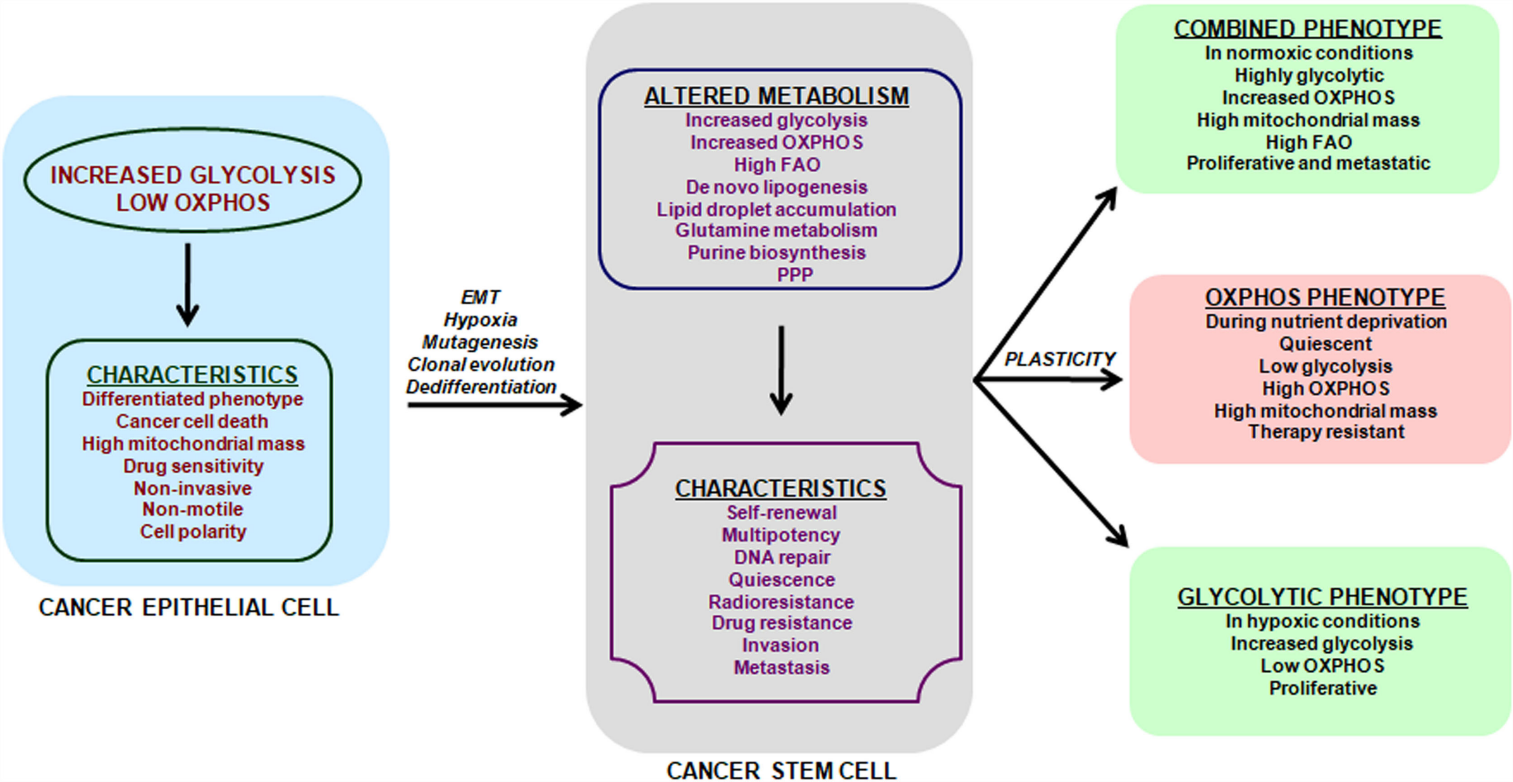
Metabolic features and plasticity of CSCs. Cancer is a heterogeneous disease with multiple sub-populations of cells and CSCs form the self-renewing and tumorigenic core in a tumor. Cancer cells have a predominant glycolytic phenotype and use aerobic glycolysis for tumor growth. This altered metabolism in cancer cells may trigger EMT, hypoxia, cell de-differentiation, mutagenesis and clonal evolution for acquisition of CSCs phenotype. The metabolic alterations in CSCs promote cell-renewal, immune system escape and invasive and metastatic potential. CSCs unlike cancer cells can use glycolysis, OXPHOS or both, depending on their oncogenic background and bio-energetic needs. This freedom of metabolic choice makes CSCs metabolically plastic and they easily shuffle between metabolic phenotypes, based on their state i.e. proliferative or quiescent. EMT, epithelial to mesenchymal transition; FAO, fatty acid oxidation; PPP, Pentose phosphate pathway.

Till date the origin of CSCs remains elusive. Two models are postulated to explain the genetic and functional heterogeneity of cancer in a single patient: the clonal evolution model and the cancer stem cell (CSC) hypothesis (19). The clonal evolution model suggests that multiple stepwise oncogenic mutations in somatic cells leads to tumor formation and natural selection favors the tumor cells with aggressive phenotype (20, 21). The CSC hypothesis suggests that metabolic events occurring in cancer epithelial cells may generate CSCs (**Figure 1**). Altered metabolic events in cancer cells may affect chromatin organization and activate epigenetic program (22) which may further fuel metabolic-reprogramming of CSCs. Two proposed models explain how metabolic alterations could affect epigenetics (22). In the first model, metabolism reprogramming facilitates differentiation of one cell type to another by altering chromatin modifications without affecting the epigenomic landscape. The second model proposes that altered metabolism induces new potential cell types *via* creation of novel stable epigenetic states, thus reshaping the entire epigenomic landscape. In this model, altered metabolism remodels chromatin by either inducing gene expression or affecting availability of substrates and cofactors for chromatin-modifying enzymes. In either case, the end-result is a novel cell state that is irreversible as epigenomic landscape has changed.

Metabolic characterization of CSCs has been a challenging task, as CSCs lack a common metabolic phenotype across cancer types. CSCs metabolic pattern differ from adult stem cells (SCs) and use either glycolysis or OXPHOS (**Figure 1**) triggering cellular plasticity in CSCs (23). Thus, an understanding of CSCs metabolic features will help target CSCs specifically and prevent cancer progression. Current review summarizes the various metabolic features of CSCs along with therapeutic interventions that can be adopted to target key energy processes in CSCs. Targeting the metabolic flexibility in CSCs can emerge as an effective strategy for preventing or minimizing disease progression and recurrence.

## Metabolic Features of CSCs

Growth factors, nutrients and oxygen in the tumor microenvironment provide necessary energy sources and growth signals for CSCs generation and proliferation. Recently, metabolism has been identified as a major component in CSCs biology, as oncogenic alterations has been observed to cause metabolite-driven dissemination of CSCs (19). Multipotent SCs use glycolysis (24, 25), have fewer mitochondria and produce less reactive oxygen species (ROS) (26, 27). Higher ROS levels cause SCs dysfunction (28–30) and shift to OXPHOS with increased ROS production leads to differentiated SCs progeny.

### Glycolysis

CSCs were hypothesized to be glycolytic (31), as SCs rely primarily on glycolysis to generate energy (32). However, CSCs are more glycolytic than SCs in various cancers (33–35). Upregulation of glycolytic genes precede pluripotency markers expression, thus switching from OXPHOS to glycolysis promotes stemness in CSCs and is not an outcome of attaining pluripotency (36). CSCs’ glucose uptake and hence lactate and ATP production is higher (37) and glycolysis inhibition or glucose starvation cause CSCs’ death (19, 38). Glycolytic CSCs are shown in CD133^+^ liver carcinoma cells (39), osteosarcoma-initiating cells (40), breast cells (41) and glioblastoma cells (35).

Glycolysis is preferred in breast CD44^+^CD24^low^EPCAM^+^ CSCs, sphere-forming radio-resistant nasopharyngeal carcinoma cells (42) and CD133^+^CD49f^+^ tumor initiating cells (TICs) in hepatocellular carcinoma (43). Elevated expression of oncogenic MYC drove stemness in these cancer types (44) and MYC-driven glycolytic program determined tumorigenic potential (45), thus making MYC a likely candidate linking glycolysis and stemness.

Lactate supports stemness by upregulation of transcription factor SP1 and increases aggressiveness, invasiveness and immune-suppression through sterol regulatory element-binding protein 1 (SREBP1) (46–51). Hypoxia-inducible factor-1 (HIF-1) promotes glycolysis in CSCs and declines OXPHOS and TCA cycle (52). HIF-1 reduces ROS production and upregulates glucose transporters (GLUT) and hexokinase (HK2) expression, pyruvate kinase (PK) activity and LDHA levels and downregulates pyruvate dehydrogenase (PDH) levels (52). HIF-1 promotes self-renewal and pluripotency in various cancers making them treatment resistant (19, 53, 54).

NANOG-expressing hepatocellular CSCs have higher glycolysis and fatty acid oxidation (FAO) rates, and lower OXPHOS and ROS generation (43). CSCs secretome have enriched levels of glycolytic and antioxidant pathways proteins and secreted high levels of ALDH than differentiated cells from colorectal tumors (24). ALDH detoxifies anticancer drugs such as maphosphamide and CSCs secreting ALDH promoted self-preservation and protected nearby differentiated mature cancer cells, leading to therapy resistance (24). Ovarian CSCs with glycolysis enrichment, *de novo* fatty acid synthesis, and decreased mitochondrial respiration and anaplerotic flux, led to aggressive tumors with therapy resistance to cisplatin in comparison to mature cancer cells (34).

### Mitochondrial Respiration

As an energy source, OXPHOS is more efficient than glycolysis, but has a slower rate to produce energy. Quiescent or slow-cycling tumor-initiating CSCs prefer OXPHOS metabolism over glycolysis (**Figure 1**), consume less glucose, have lower lactate and higher ATP levels (55–57). OXPHOS-dependent CSCs with low glycolytic reserves are shown in acute myeloid leukemia, CD133^+^ glioblastoma, melanoma, pancreatic and ovarian cancer (58–63). In breast CSCs, elevated OXPHOS levels trigger chemotherapeutic resistance through synergistic action of MYC and MCL1 (64).

CSCs using OXPHOS have higher mitochondrial mass with increase in membrane potential and rates of oxygen consumption (62, 65, 66). Mitochondrial mass is a vital metabolic biomarker of CSCs (65, 67). Tumor cells without mitochondrial DNA (mtDNA) grew slowly and acquisition of mtDNA from host cells led to tumor-initiation and drug resistance in these tumor cells (68), suggesting mitochondrial function as a target for CSCs treatment. Master mitochondrial biogenesis regulator, peroxisome proliferator-activator 1 alpha (PGC1α) maintained stemness characteristics (69) in breast cancer (70) and pancreatic CD133^+^ CSCs (66) and increased chemoresistance in CSCs (64, 71–73). NANOG is a pluripotency gene that supports tumorigenesis through OXPHOS and fatty acid metabolism (43). Some breast CSCs show elevated glucose consumption and ATP production, higher mitochondrial activity but lower lactate levels, suggesting that OXPHOS and glycolysis may not be mutually exclusive to CSCs (62).

### Glutamine Metabolism

Glycolysis and OXPHOS may not completely support CSCs metabolism, thus glutamine compensates for glucose shortage (74, 75). Although a non-essential amino acid, glutamine becomes essential for cancer cells (76) and CSCs from lung, pancreatic and ovarian cancer have shown glutamine dependence (77, 78). CSCs rely on glutamine for carbon and amino-nitrogen for protein, nucleotide and lipids biosynthesis (79). Glutamine metabolism is rewired by mutations in mitochondrial DNA (mtDNA) (80) and oncogenic alterations in KRAS (81, 82) and c-Myc (83) in tumor cells. Glutamine metabolism in c-Myc-over-expressing cells suggests a pluripotency gene profile dependence on glutamine (84). In pancreatic CSCs, glutamine unavailability reduced stemness characteristics and increased radiation therapy sensitivity (77). L-DON (a glutamine analog) inhibited glucose metabolism and prevented systemic metastasis to liver, lung and kidney in mice (85).

### Lipid Metabolism

Cells use an anabolic process of fatty acid synthesis (FAS) to derive energy from fatty acid metabolism for cell growth and proliferation, and a catabolic process of fatty acid oxidation (FAO) for NADH and ATP production (86). CSCs are extremely reliant on *de novo* lipid biosynthesis, lipid oxidation and lipid metabolizing enzymes (87, 88).

Lipid accumulation correlates with tumor stage in mice with prostate cancer (89). *De novo* lipid synthesis associated transcription factor, SREBP-2 activated c-Myc transcription in prostate cancer, enhancing CSCs properties (90). Increased lipid droplet content in colorectal CSCs (91), upregulated lipogenesis in glioma (92) and pancreatic cancer CSCs (93), and increased fatty acid oxidation (FAO) in breast cancer (94) and leukemic cells (95) maintained stemness. High levels of unsaturated lipids in ovarian CSCs promotes cancer stemness and tumor initiation capacity (96).

CSCs use mitochondrial FAO for ATP and NADPH generation to survive loss of matrix attachment (97, 98). Pluripotency factor NANOG-induced FAO genes expression promoted chemoresistance in TICs in hepatocellular carcinoma (43). Hematopoietic stem cells (HSCs) and leukemia-initiating cells depend on FAO for self-renewal (95, 99) and thus FAO inhibition is a potential pharmacological opportunity to target CSCs (98). Lipid metabolism enzymes, ACSVL3 (acyl-CoA synthetase very-long-chain 3) and ALOX5 (arachidonic acid 5-lipoxygenase) promoted glioblastoma CSCs self-renewal and tumorigenicity (100, 101).

### Other Metabolic Features

Mutations in isocitrate dehydrogenase (IDH1 and 2) promote stem-ness in leukemia by aberrant conversion of α-ketoglutarate (αKG) to an analogue named 2-hydroxyglutarate (2-HG). Intracellular accumulation of 2-HG promoted a pro-leukemic phenotype by inhibiting tet methylcytosine dioxygenase 2 (TET2) function, increased self-renewal and impaired differentiation of hematopoietic SCs (102–104).

Elevated purine synthesis promoted stemness in brain tumor initiating cells (BTICs) and correlated with significantly poorer overall survival in glioblastoma patients (105). MYC regulates purine synthesis enzymes and its liaison with *de novo* purine synthesis mediated selective dependence of BTICs on glucose-sustained anabolic metabolism. Inhibition of purine synthesis prevented BTICs growth by inhibiting their self-renewal capacity, but differentiated glioma cells remained unaffected (105). Thus frailty of purine synthesis in CSCs makes it a potential therapeutic target,

Lysine catabolism promoted self-renewal of CD110^+^ colorectal cancer tumor-initiating cells (TICs) by generating acetyl-CoA. Acetyl-CoA triggered LDL receptor-related protein 6 (LRP6) acetylation and phosphorylation, and finally activation of WNT signaling (106). Lysine catabolism promoted drug-resistance and metastasis to liver in CD110^+^ TICs by glutamate and glutathione synthesis, which modulated the redox status (106). Collectively, CSCs use an array of metabolism alterations to fuel their self-renewal, thus making these metabolic dependencies open to targeted therapies.

## Clinical Implications

CSCs have both distinct and flexible metabolic phenotypes between glycolysis and OXPHOS-dependent. Despite limited clinical evidence, targeting CSCs through selective metabolic modulation is an effective and promising avenue for cancer treatment. In our view, synergistic treatments using a standard cytotoxic agent and a metabolic-based therapy will improve eradication of CSCs. **Table 1** lists the available metabolic targeting agents undergoing clinical trials in various cancers.

**Table 1:** Clinical trial status of drugs targeting metabolic pathways.

METABOLIC PATHWAYS	TARGET MOLECULE	DRUG	CANCER TYPE	CLINICAL TRIAL PHASE	RECRUITMENT STATUS	CLINICAL TRIAL NUMBER
**Amino acid metabolism**	Glutaminase	**Phenylacetate**	Brain tumor	Phase-II	Completed	NCT00003241
		**CB-839**	Renal Cell Carcinoma	Phase-II	Active	NCT03428217
			Hematological tumor	Phase-I	Completed	NCT02071888
			Leukemia	Phase-I	Completed	NCT02071927
	Asparagine	**Asparaginase**	Acute myloid leukemia	Phase-III	Active	NCT00369317
				Phase-III	Recruiting	NCT02521493
		**Pegylated L-Asparaginase**	Epithelial Ovarian Cancer, Fallopian Tube Cancer, and/or Primary Peritoneal Cancer	Phase II	Completed	NCT01313078
	Arginine	**Arginine deiminase**	Soft Tissue Sarcoma, Osteosarcoma, Ewing’s Sarcoma, and Small Cell Lung Cancer	Phase-II	Active	NCT03449901
**Fatty acid synthesis**	FASN	**TVB-2640**	Colon Cancer	Phase-I	Recruiting	NCT02980029
			Solid Malignant Tumor	Phase-I	Completed	NCT02223247
			Breast Cancer	Phase-II	Recruiting	NCT03179904
			Non-Small Cell Lung Carcinomas	Phase-II	Recruiting	NCT03808558
			Astrocytoma	Phase-II	Active, not recruiting	NCT03032484
**Cholesterol synthesis**	HMGCR	**Statins**	Breast Cancer	Phase-III	Recruiting	NCT03971019
			Prostate Cancer	NA	Completed	NCT01428869
			Gastric Cancer	NA	Completed	NCT01813994
			Breast Cancer	Phase-II	Completed	NCT00816244
**Lipid-Mediated Signaling**	Prostaglandin-endoperoxide synthase 2	**Celecoxib**	Breast Cancer	Phase-III	Completed	NCT02429427
			Pancreatic Cancer	Phase-II	Completed	NCT00068432
			Lung Cancer	Phase-II	Completed	NCT00030407
	EP4 receptor (prostaglandin receptor)	**PGE1**	Prostate Cancer	Phase-II	Completed	NCT00080808
			Penile Cancer	NA	Completed	NCT00955929
		**Omega-3 polyunsaturated fatty acids (ω-3 PUFAs)**	Skin Cancer	NA	Completed	NCT01032343
			Bladder Cancer	NA	Recruiting	NCT04664816
			Breast Cancer	NA	Active, not recruiting	NCT02295059
**Tricarboxylic acid cycle (TCA) Cycle**	Pyruvate dehydrogenase kinase (PDK1)	**Dichloroacetate (DCA)**	Head and neck cancer	Phase-I	Completed	NCT01163487
			Glioblastoma and Other Recurrent Brain Tumors	Phase-I	Completed	NCT01111097
			Brain cancer	Phase-II	Completed	NCT00540176
			Metastatic solid tumor	Phase-I	Completed	NCT00566410
**Glycolysis**	GLUT4	**Ritonavir**	HER2-expressing Advanced Solid Malignant Tumors	Phase-I	Active	NCT03383692
	Hexokinase	**2-deoxy-D-glucose (2-DG)**	Lung cancer	Phase-III	Active	NCT01394679
			Prostate cancer	NA	Active	NCT00002981
	Pyruvate kinase (PK)	**TLN-232**	Renal Cell Carcinoma	Phase-II	Completed	NCT00422786
**OXPHOS**	Cytochrome b	**Atovaquone**	Acute myloid leukemia	Phase-I	Active	NCT03568994
			Lung cancer	Phase-I	Recruiting	NCT04648033
	Respiratory complex I, mitochondrial glycerol-3-phosphate dehydrogenase (mGPDH)	**Metformin**	Breast cancer	Phase-II	Active	NCT02028221
			Prostate cancer	Phase-II	Active	NCT02945813
			Endometrial cancer	Phase-II	Active	NCT02755844
			Lung cancer	Phase-II	Active	NCT03048500
**Pentose phosphate pathway (PPP)**	Glucose-6-phosphate dehydrogenase (G6PDH)	**Resveratrol**	Colon cancer	Phase-I	Completed	NCT00256334
			Gastrointestinal Tumors	NA	Completed	NCT01476592
			Colorectal Cancer	Phase-I	Completed	NCT00433576
			Colorectal Cancer	Phase-I	Completed	NCT00920803
	G6PDH and ribose-5-phosphate (R-5P)	**Dehydroepiandrosterone (DHEA)**	Vaginal Atrophy In Breast Cancer Survivors	Phase-IV	Recruiting	NCT04705883
			Multiple myeloma	Phase-II	Completed	NCT00006219
	G6PDH, 6PGDH and Transaldolase TA	**Arginine and ascorbic acid combination**	Head and Neck Cancer	NA	Completed	NCT03531190
**Nucleotide biosynthesis**	DNA & RNA synthesis	**5-Fluorouracil (5-FU)**	Pancreatic cancer	Phase-II	Active	NCT02352337
			Colon cancer	Phase-I	Active	NCT02724202
			Biliary tract cancer	Phase-II	Active	NCT03524508
			Bladder cancer	Phase-II	Active	NCT00777491
		**Cytarabine**	Multiple myeloma	Phase-II	Active	NCT02416206
		**Methotrexate**	Head and Neck Cancers	Phase-II	Active	NCT03193931
			Breast Cancer	NA	Completed	NCT00615901
			Head and Neck Cancer	Phase-III	Active	NCT01884623
			Brain Tumors	Phase-I	Completed	NCT02458339
	DNA synthesis	**Folate**	Colorectal Cancer	Phase-I	Completed	NCT00096330
			Non-Small Cell Lung Cancer	Phase-II	Completed	NCT00609518
			Head and Neck Squamous Cell Cancer	Phase-II	Completed	NCT01183065
	Methyltransferases	**Genistein**	Breast cancer	Phase-II	Completed	NCT00244933
			Prostate cancer	Phase-II	Completed	NCT00584532
			Colorectal cancer	Phase-I	Completed	NCT01985763
			Pancreatic cancer	Phase-II	Completed	NCT00376948
		**Epigallocatechin Gallate (EGCG)**	Colorectal Cancer	Phase-I	Recruiting	NCT02891538
			Lung Cancer	Phase-II	Enrolling by invitation	NCT02577393
	Histone deacetylases (HDAC)	**Butyrate**	Rectal cancer	Phase-II	Completed	NCT04795180
		**Sulforaphane**	Prostate cancer	Phase-II	Completed	NCT01228084
		**3,3 Diindolylmethane**	Prostate Cancer	Phase-II	Completed	NCT00888654
	Histone acetyltransferase	**Curcumin**	Colon cancer	Phase-I	Active	NCT02724202
			Breast cancer	Phase-II	Completed	NCT01042938
			Prostate cancer	Phase-II	Active	NCT02724618
			Colon cancer	Phase-I	Active	NCT02724202
			Gastric cancer	Phase-II	Active	NCT02782949
	Acetylation of non-histone proteins	**Butyrate**	Epstein Barr virus-induced malignancies	Phase-I	Completed	NCT00006340
**Combination treatments**	mTOR (mammalian target of rapamycin)	**Everolimus**	Pancreatic cancer	Phase-II	Active, not recruiting	NCT02294006
	Respiratory complex I, mGPDH	**Metformin**				
	Cyclooxygenase (COX)	**Aspirin**	Colorectal Cancer	Phase-II	Unknown	NCT03047837
	Respiratory complex I, mGPDH	**Metformin**				
	HMG-CoA reductase	**Atorvastatin**	Triple Negative Breast Cancer	Phase-II	Recruiting	NCT03358017
	Farnesyl pyrophosphate (FPP) synthase	**Zoledronate**				
	Arginase	**INCB001158**	Advanced/Metastatic Solid Tumors	Phase-I	Active, not recruiting	NCT02903914
	Programmed cell death protein 1 (PD-1)	**Pembrolizumab**		Phase-II		
	Glutaminase	**CB-839**	Solid Tumors	Phase-I	Terminated	NCT03875313
	Poly ADP ribose polymerase (PARP)	**Talazoparib**		Phase-II	(Slow Enrollment)	
	Respiratory complex I, mGPDH	**Metformin**	Prostate Cancer	Phase-II	Completed	NCT01796028
	Microtubules	**Taxotere**				
	Respiratory complex I, mGPDH	**Metformin**	Cancer	Phase-III	Not yet recruiting	NCT02201381
	HMG-CoA reductase	**Atorvastatin**				
	30S ribosomal subunit	**Doxycycline**				
	Tubulin	**Mebendazole**				
	Coenzyme A	**Coenzyme A**	Castration-resistant Prostate Cancer	Phase-I	Recruiting	NCT04839055
	CYP17A1 (17 alpha-hydroxylase/C17,20 lyase)	**Abiraterone**		Phase-II		
	Respiratory complex I, mGPDH	**Metformin**	Endometrial Cancer	Phase-II	Active, not recruiting	NCT02755844
	PARP	**Olaparib**		Phase-I		
	Phosphoramide mustard	**Metronomic cyclophosphamide**				
	Respiratory complex I, mGPDH	**Metformin**	Colorectal Cancer	Phase-II	Completed	NCT01941953
	DNA & RNA synthesis	**Fluorouracil**	Pancreatic Cancer	Phase-II	Completed	NCT01666730
	Respiratory complex I, mGPDH	**Metformin**	Breast Cancer	Phase-II	Completed	NCT01310231
	Athracyclines, platinum, taxanes or capecitabine; first or second line	**Standard chemotherapy**				
	Respiratory complex I, mGPDH	**Metformin Hydrochloride**	Endometrial Cancer	Phase-II	Active, not recruiting	NCT02065687
	DNA	**Carboplatin**				
	Respiratory complex I, mGPDH	**Metformin**	Liver Cancer	Phase-III	Unknown	NCT03184493
	Prostaglandin-endoperoxide synthase 2	**Celecoxib**	Prostate Cancer	Phase-II & III	Recruiting	NCT00268476

### Targeting Glycolysis

Glycolytic CSCs can be targeted for glycolytic enzymes (hexokinase (HK), phosphoglycerate kinase, pyruvate kinase) and glucose transporters (GLUT1-4). Direct inhibition of GLUTs results in a total disruption of glucose uptake and hence energy metabolism, and GLUT inhibitors such as phloretin, fasentin and WZB117 have shown anticancer effects in preclinical models (107–110). However, ubiquitous expression of GLUTs even in normal cells challenges the explicit inhibition of CSCs glucose uptake and leads to side-effects.

HK enzymes catalyze the first step of glycolysis and their inhibition *via* 2-deoxy-D-glucose (2-DG), benserazide, lonidamine (LN) and genistein-27 (GEN-27) are being used for cancer treatment (111–114). 2-DG is a synthetic analog of glucose that competitively inhibits glucose transport (115) and can be used in combination with cisplatin/docetaxel as an anti-cancer agent (116, 117). 2-DG inhibited glycolysis and CSCs phenotype in triple-negative breast cancer cells (118) and 2-DG with biguanides (such as 3-bromopyruvate, 3-BP) prevented colon cancer cell proliferation (119).

Pyruvate is converted into mitochondrial acetyl-CoA in the cytosol and is negatively regulated by pyruvate dehydrogenase kinase (PDK) enzyme. This shifts cellular metabolism from OXPHOS to glycolysis and thus targeting PDK can inhibit cellular proliferation of CSCs. Dichloroacetate (DCA) activates mitochondrial pyruvate dehydrogenase (PDH) by inhibiting PDK (120), is fairly well-tolerated with fewer side effects and is being tested in several anticancer clinical trials (121, 122).

CSCs can oscillate between metabolic phenotypes during oxygen deprivation and glucose starvation, and thus targeting mechanisms underlying these metabolic adaptations can effectively eliminate CSCs. Hypoxia-inducible factors (HIFs) promote tumor progression in response to localized hypoxia by switching to glycolysis from OXPHOS, activating Notch pathway and expression of Oct4 transcription factor (123, 124). This suggests HIF-1α’s role in self-renewal and multipotency and targeting HIFs can be a prospective treatment for CSCs. Metformin, although an antidiabetic drug, attenuated glycolysis flux in hepatocellular carcinoma cells (125) and improved radiotherapy response in prostate and colon cancer tumor xenograft models (126). Epigallocatechin gallate (EGCG) is an inhibitor of glycolysis and its co-treatment with gemcitabine enhanced pancreatic cancer cell death both *in vitro* and in xenografts (127).

### Targeting Mitochondrial Respiration

Several OXPHOS-targeting pharmacological agents are being explored in clinical trials for cancer treatment (**Table 1**) and have potential to target CSCs. OXPHOS inhibition overcame drug resistance in slow-cycling melanoma cells and mitochondria-targeted antibiotics prevented sphere formation and tumorigenesis in CSCs (61, 128). Metformin inhibited mitochondrial electron transport chain complex I and diminished OXPHOS (129). Metformin caused energy emergency and hence apoptosis in OXPHOS-dependent pancreatic cancer stem cells (CSCs), but spared their glycolytic differentiated progenies (66). Diabetic patients receiving metformin have a lower mortality rate from cancer and hence a better prognosis (130, 131). Phenformin, a biguanide formerly used in diabetes and a mitochondrial inhibitor induced non-small cell lung cancer (NSCLC) cells apoptosis (132).

CSCs mitochondrial mass and metabolism can be targeted using approved antibiotics like tetracyclines, salinomycin and erythromycins. Antibiotic salinomycin inhibits OXPHOS (133) and salinomycin treatment reduced breast CSCs gene expression. Antibiotic tigecycline inhibited mitochondrial translation in mitochondrial associated ribosomes in OXPHOS-dependent leukemia cells (134).

CSCs using OXPHOS have a higher mitochondrial membrane potential (Δψm) and thus Δψm can be explored for selective accumulation of cytotoxic drugs. Triphenylphosphonium (TPP) accumulates in the mitochondrial matrix (135) and conjugation of TPP to doxorubicin prevented drug efflux by enhancing drug selectivity in cancer cells (136). Dual inhibition of glycolysis and OXPHOS in sarcoma cells, using 2-DG and oligomycin/metformin co-treatment (137), suggests that simultaneous inhibition of glycolytic and mitochondrial respiration is more effective to eradicate CSCs (138, 139).

### Targeting Glutamine Metabolism

Although a non-essential amino acid, glutamine becomes essential as a favored respiratory fuel for cancer cells and thus depriving glutamine is a potential anti-cancer strategy. Glutamine metabolism can be blocked by inhibiting glutaminase 1 (GLS1), an enzyme that converts glutamine to glutamate. GLS1 inhibition disrupted redox balance in CSCs and sensitized lung and pancreatic cancers to radiotherapy (77, 140). GLS1 inhibitors, BPTES (141) and CB-839 reduce intracellular glutamate and 2-hydroxyglutarate (an oncometabolite) levels. Lower glutamate levels inhibited cell growth, induced apoptosis and differentiation in Acute Myeloid Leukemia (AML) cells (142). CB-839 is under clinical trials for various cancers including renal cell carcinoma, hematologic cancer and leukemia (**Table 1**).

### Targeting Lipid Metabolism

Cancer cells predominately use glycolysis for ATP production instead of oxidizing energy-rich substrates. However, unlike non-cancerous cells dependence on dietary lipids, cancer cells use *de novo* lipogenesis. Thus targeting fatty acid synthase (FASN), a central enzyme to lipogenesis, is a promising strategy to eliminate CSCs. FASN inhibitor cerulenin reduced *de novo* lipogenesis and in turn proliferation, migration and stemness of glioma stem cells (GSCs), induced apoptosis in colon cancer cell lines (92, 143) and blocked proliferation of pancreatic spheres (93). C75 decreased HER2+ breast cancer cells self-renewal capacity at non-cytotoxic concentrations (144). However, due to toxicity issues in *in-vivo* studies owing to high selectivity of FASN inhibitors, only one FASN inhibitor (TVB-2640) is under clinical trials to date (**Table 1**).

Studies show that increased fatty acid production in cancer cells raises their dependence on desaturases (enzymes that add double bonds into acyl-CoA chains). Thus targeting desaturase enzyme activity may provide a novel approach to selectively interfere lipid metabolism in CSCs. Several stearoyl-CoA desaturase-1 (SCD-1) inhibitors have effectively targeted stemness in pre-clinical models of cancer. Inhibitors like CAY10556 and SC-26196 reduced stem-ness markers and inhibited *in-vitro* sphere formation and *in-vivo* tumorigenicity, by down-regulating Hedgehog and Notch expression in aldehyde dehydrogenase (ALDH)- and CD133-enriched ovarian cells and had no effect on differentiated cells (96). Similarly, SCD-1 inhibitors (SSI-4 or A939572) promoted differentiation in chemo-resistant hepatospheres with little toxicity *in vivo* (145). MF-438 reduced expression of self-renewal and pluripotency markers in lung ALDH1^+^ cells (146).

Along with *de novo* lipogenesis, cancer cells also take lipids from the extracellular milieu (147) using LDL receptor (LDLR) (148), CD36 fatty acid translocase, fatty acid transport proteins (FATPs) (149) or fatty acid-binding proteins (FABPs) (150). Inhibition of CD36 transporter with 2-methylthio-1,4-naphtoquinone reduced self-renewal and promoted apoptosis in CD133^+^ glioblastoma (151) and sulfosuccinimidyl oleate reduced chemo-resistant leukemic stem cells (152). CD36-neutralizing antibodies inhibited progression and metastasis of oral squamous cell carcinoma and had no reported toxicity *in-vivo* (153).

Highly proliferating cells also have a higher demand for components of cell membrane like cholesterol. Cholesterol is either taken up from exogenous sources or synthesized using FASN or mevalonate pathway (154). Statins inhibit cholesterol synthesis through the mevalonate pathway and their target enzyme is 3-hydroxy-3-methyl-glutharyl-coenzyme A reductase (HMGCR). Statins treatment decreased CSCs self-renewal capacity and number in breast (155), nasopharyngeal (156) carcinomas and CD133^+^ brain TICs (157). MYC controls over-expression of mevalonate pathway genes and thus anti-CSCs effects of statins could be due to MYC inhibition (157).

Synthesized or accumulated fatty acids are also converted to signaling lipids and energy *via* FAO, in addition to membrane incorporation or being stored. FAO is an essential energy source in non-glycolytic tumors (158, 159), as CSCs show higher FAO in nutrient-deprived conditions (63, 86, 160, 161). FAO promotes pluripotency and chemoresistance (94) by reducing ROS production (162, 163) and promoted metastatic capacity in sphere-derived cells (164). Etomoxir, an inhibitor of FAO, inhibited mammosphere formation in hypoxic breast CSCs (165) and eradicated half of quiescent leukemia SCs (99), suggesting that FAO inhibitors hinder CSCs survival. In hepatocellular carcinoma, etomoxir sensitized CSCs to sorafenib treatment (43). Soraphen A, cerulenin and resveratrol inhibited FAO and lowered stemness markers and spheroid formation in CSCs (92, 166, 167).

Lipids also support CSCs functionality by being second messengers in signal transduction pathways. Sphingolipids, eicosanoids (prostaglandin E2) and glycerophospholipids (lysophosphatidic acid (LPA)) boost CSCs number by activation of Notch, AKT and NF-kB pathways in breast, bladder, colorectal (CRC) and ovarian cancer (168–171). Lipid-mediated signaling in CSCs thus can be targeted using inhibitors and dietary supplements. Inhibition of autotoxin (ATX) (a lysophosphatidic acid (LPA)-producing enzyme) with S32826 or PF8380 reduced tumorigenicity and chemoresistance *in-vivo* (171). Inhibition of LPA production in cancer cells modulated the immune system by inducing monocytes differentiation to macrophages and launching cancer-associated fibroblasts (CAFs) phenotype (172, 173). Prostaglandins are major lipid mediator in CSCs and celecoxib treatment of Apc^Min^/þ mice reduced number of CD133^+^CD44^+^ cells and tumor burden (170). Celecoxib reduced patient-derived CSCs content and liver metastatic tumors number in NOD scid gamma (NSG) mice and weakened chemoresistance in bladder carcinomas, indicating its potential as an adjuvant therapy (169). In contrast, reduction of CD34^+^ cells in chronic myelogenous leukemia (CML) xenograft model by EP4 receptor (prostaglandin receptor) agonist misoprostol or PGE1 (FDA-approved), suggests a context-dependent role of prostaglandins in stem-ness (174). Further, dietary omega-3 polyunsaturated fatty acids (ω-3 PUFA) decreased CRC risk and reduced CD133^+^ content in CRC cell lines (175, 176). Eicosapentaenoic acid (EPA) and docosahexaenoic acid (DHA) supplementation decreased breast tumorspheres proliferation (177) and EPA with chemotherapy suppressed tumor growth in mice (178), suggesting an anti-CSCs properties of ω-3 PUFAs.

### Combination Treatments

CSCs can also attain a combined metabolic phenotype where both glycolysis and OXPHOS are utilized (**Figure 1**). This phenotype can be attained by direct association of AMP-activated protein kinase (AMPK, master regulator of OXPHOS) and HIF-1 (master regulator of glycolysis) activities (179). High AMPK/HIF-1 activities leads to higher glycolysis and OXPHOS, and provide enhanced proliferation and clonogenicity compared to only glycolytic or OXPHOS phenotype (179). In addition, CSCs metabolize glutamine along with glucose for carbon and amino-nitrogen to synthesize amino acids, nucleotides and lipids (79). Additionally, CSCs also use *de novo* lipogenesis to increase their bioenergetic requirements and are linked in tumor metastasis (88). Also preclinical and clinical setting has shown that targeting a single metabolic pathway like glycolysis has low success rates and enhanced side effects as GLUT transporters are ubiquitous. Also, inhibition of hexokinase II with ionidamine showed no significant improvement in overall survival but led to elevated toxicity (114, 180–182). Thus combination treatments targeting two or more metabolic pathways will majorly erase CSCs, prevent tumor relapse and prevent side-effects of a single treatment.

Further, combining a standard cytotoxic therapy with a metabolic inhibitor will probably enhance CSCs eradication. Combinations of metformin and JQ-1 (bromodomain and extraterminal motif (BET) inhibitor) in pancreatic cancer (66) or PI3K inhibitor in ovarian cancer (183) blocked both OXPHOS and glycolysis. Apart from direct metabolic inhibition, targeting oncogenes regulating cellular metabolism will also eradicate CSCs effectively. KRAS mutation occurs in about 90% of pancreatic cancer cases (184) and KRAS drives glycolysis and nucleic acids synthesis (185, 186). c-MYC is essential for glycolysis in cancer (187, 188) and MYC suppression prevents mitochondrial inhibitors resistance (66, 75). Thus combination approaches can be extended to target CSCs as an anti-cancer strategy. **Table 1** lists the clinical trials using combination treatments for various cancers.

## Future Challenges

**Figure 1** summarizes the known CSCs’ metabolic phenotypes and how these phenotypes switch with metabolic stressors like nutrient deprivation and hypoxia. However, melanoma cells attain a drug-tolerant “idling state” after enduring MAPK inhibition (MAPKi) and this state has a metabolically Low/Low (L/L) phenotype, where both AMPK/HIF-1 activity and OXPHOS/glycolysis are minimal (189). L/L phenotype does not favor tumorigenicity but supports cell division. These idle L/L drug-tolerant cells accumulate mutations to promote relapse post MAPKi melanoma treatment (189).

Further adding to the complexity of CSCs metabolism, Luo et al. (190) showed that breast cancer stem cells (BCSCs) have two states: quiescent mesenchymal-like (M) and proliferative epithelial-like (E). Proliferative E-BCSCs showed higher mitochondrial OXPHOS, whereas M-BCSCs have enrichment of glycolysis and gluconeogenesis pathways and hypoxia promotes M to E transition in BCSCs (190). Thus CSCs’ multiple metabolic phenotypes (glycolytic, OXPHOS, combined and L/L) explain the futility of current efforts to eradicate CSCs and a deeper understanding of CSCs metabolic plasticity would translate to better therapeutic strategies.

## Concluding Remarks

CSCs provide treatment resistance and promote metastasis during tumor growth and targeting metabolism holds potential in overcoming cancer recurrence and metastasis by CSCs. Deciphering metabolic reprogramming in cancer showed differences between metabolic phenotypes of CSCs and their differentiated counterparts. CSCs metabolism shuffles between glycolysis and OXPHOS primarily, however the mechanisms of CSCs metabolic heterogeneity are still unknown. Current knowledge suggests that carefully designed metabolic therapies have potential to be more effective against CSCs. Further, co-targeting CSCs using metabolic drugs and traditional anticancer treatments could be more efficient. The ongoing clinical trials targeting CSCs show a promising future for cancer therapy and are worth exploring further. More preclinical and clinical studies are thus required to uncover novel metabolic targets in CSCs.

## Author Contributions

SB conceptualized the article and reviewed the literature, JK did literature search and drafted the manuscript. Both SB and JK contributed to the final version.

## Conflict of Interest

The authors declare that the research was conducted in the absence of any commercial or financial relationships that could be construed as a potential conflict of interest.

## Publisher’s Note

All claims expressed in this article are solely those of the authors and do not necessarily represent those of their affiliated organizations, or those of the publisher, the editors and the reviewers. Any product that may be evaluated in this article, or claim that may be made by its manufacturer, is not guaranteed or endorsed by the publisher.
